# Crystal structure and Hirshfeld surface analysis of 4-bromo-2-chloro­phenyl (*E*)-3-[4-(undec­yloxy)phen­yl]acrylate

**DOI:** 10.1107/S2056989025007078

**Published:** 2025-08-15

**Authors:** H. C. Devarajegowda, B. S. Palakshamurthy, H. Anil Kumar, H. T. Srinivasa, M. Harish Kumar

**Affiliations:** a Department of Physics Yuvaraja's College University of Mysore,Mysore 570005 Karnataka India; b Department of PG Studies and Research in Physics Albert Einstein Block UCS Tumkur University, Tumkur Karnataka-572103 India; c Department of Physics, Government First Grade College, Chikkabalapura, Karnataka-562101, India; d Raman Research Institute, C V Raman Avenue Sadashivanagar Bangalore Karnataka-560080 India; Universidade Federal do ABC, Brazil

**Keywords:** crystal structure, 4-bromo-2-chloro­phen­yl, Hirshfeld surface

## Abstract

In the title compound, C_26_H_32_BrClO_3_, the dihedral angle between the 4-bromo-2-chloro­phenyl ring and the aromatic ring of (alk­yloxy)phenyl moiety is found to be 77.21 (2)°. The torsion angle associated with the ester moiety is 173.2 (2)° which is *anti-periplanar*. In the crystal, inter­molecular C—H⋯O hydrogen bonding links the mol­ecules into cyclic hydrogen-bonded inversion dimers with *R*^2^_2_(10) motifs. The mol­ecular structure is associated with an inversion centre and connected through two symmetrical C—H⋯O inter­actions, forming *R*^2^_2_(10) inversion dimer motif. The packing is further consolidated by C—H⋯π and C—Cl⋯π inter­actions. Hirshfeld surface analysis showed that the most significant contributions are from H⋯H (54.0%), C⋯H/H⋯C (15.2%), Br⋯H/H⋯Br (10.9%), O⋯H/H⋯O (7.8%) and Cl⋯H/H⋯Cl (2.6%) contacts.

## Abstract 2

The title was synthesized by an acid–phenol coupling reaction. In the crystal, inter­molecular C—H⋯O hydrogen bonding links the mol­ecules into cyclic hydrogen-bonded inversion dimers with *R*^2^_2_(10) motifs. The packing is further consolidated by C—H⋯π and C—Cl⋯π inter­actions.

## 1. Chemical context

Compounds based on the 4-bromo-2-chloro­phenyl scaffold are found to exhibit potent *in vitro* inhibitory activity against *Plasmodium falciparum*, making them promising candidates as transmission-blocking agents for malaria treatment (Vallone *et al.*, 2018 ▸; Kos *et al.*, 2022 ▸). The incorporation of halogen substituents in the phenyl ring is known to enhance anti­microbial activity in such mol­ecules (Radwan *et al.*, 2014 ▸). Acrylate derivatives have demonstrated anti­tumor potential by inhibiting tubulin polymerization at the cellular level (Pieters *et al.*, 1999 ▸; Jung *et al.*, 2019 ▸). The presence and prolongation of alkyl groups in the various drug mol­ecules are found to enhance the penetration of the compounds into cells, which make the mol­ecules efficient as drugs. In this context, it is found that caffeic acid phosphanium salt combined with alkyl chains acquire anti­cancer properties (Lukáč *et al.*, 2024 ▸), whereas cinnamic acid-based mol­ecules coupled with alkyl groups exhibit anti-tuberculosis activity (De *et al.*, 2011 ▸). Furthermore, increasing the alkyl chain length on amido functional groups has been shown to improve the anti-inflammatory activities of certain drugs (Matta *et al.*, 2020 ▸). Studies on 4-bromo­phenyl piperidine derivatives revealed strong binding affinities towards the COVID-19 main protease, indicating potential anti­viral activities (Lorin *et al.*, 2024 ▸). Similarly, chloro­phenyl derivatives have demonstrated inhibitory effects against the SARS-CoV-2 main protease. Despite the promising pharmacological potential, limited research has been conducted on the medicinal significance of 4-bromo-2-chloro­phenyl scaffolds. In view of this gap, we aimed to design and synthesize a novel series of compounds incorporating this moiety. Herein, we present the synthesis and characterization of the title compound, constructed by coupling the 4-bromo-2-chloro­phenyl unit with a (undec­yloxy)phenyl fragment through an ester linkage.
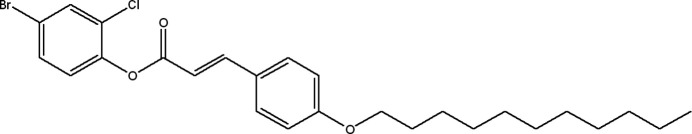


## 2. Structural commentary

**Figure 1:**
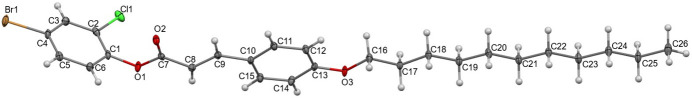
The title mol­ecule with the atom-labelling scheme and 50% probability displacement ellipsoids.

The title compound (Fig. 1 ▸) crystallizes in the space group *P*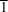. The mol­ecule is nearly planar with an r.m.s deviation of 0.076 Å. The dihedral angle between the mean planes of the 4-bromo-2-chloro­phenyl ring and the aromatic ring of the (alk­yloxy)phenyl moiety is 77.21 (2)°. With respect to the alkyl chain (O3–C26), the dihedral angle formed with the 4-bromo-2-chloro­phenyl ring is 66.94 (2)°, while that with the phenyl ring is 10.59 (2)°, indicating a more coplanar orientation between the alkyl chain and the phenyl ring. The torsion angle associated with the ester moiety (C1—O1—C7—C8) is 173.1 (2)°, which is *anti-periplanar*. The mol­ecule does not show any significant deviations in bond distances or angles.

## 3. Supra­molecular features

**Figure 2:**
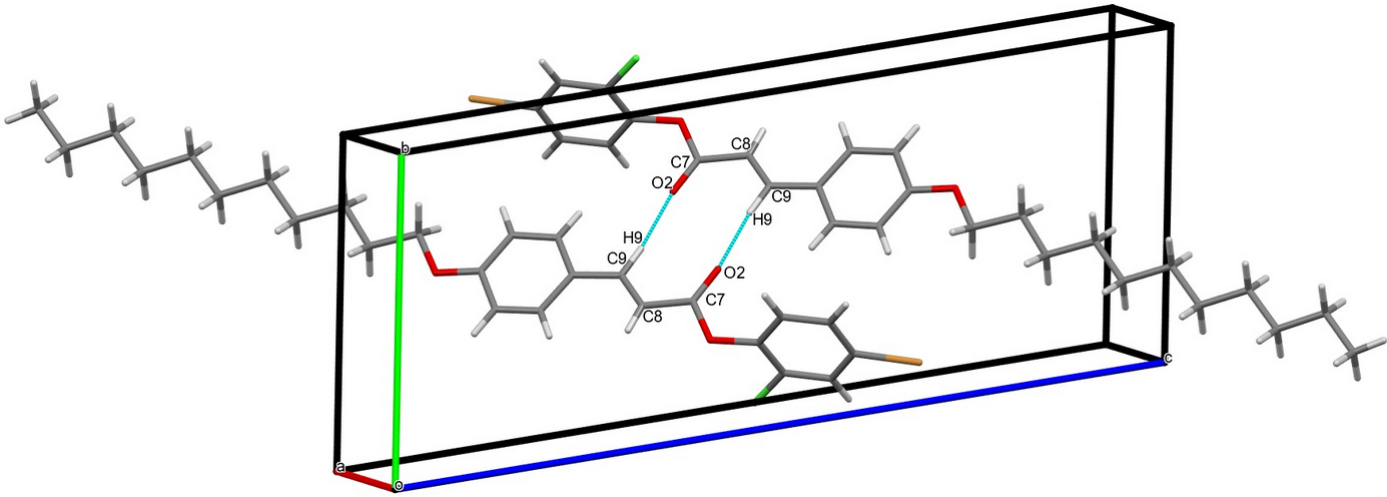
The mol­ecular packing with inter­molecular C—H⋯O inter­actions depicted by dashed pale- green coloured lines. Symmetry code: (i) −*x* − 1, −*y* + 1, −*z* + 1.

**Figure 3:**
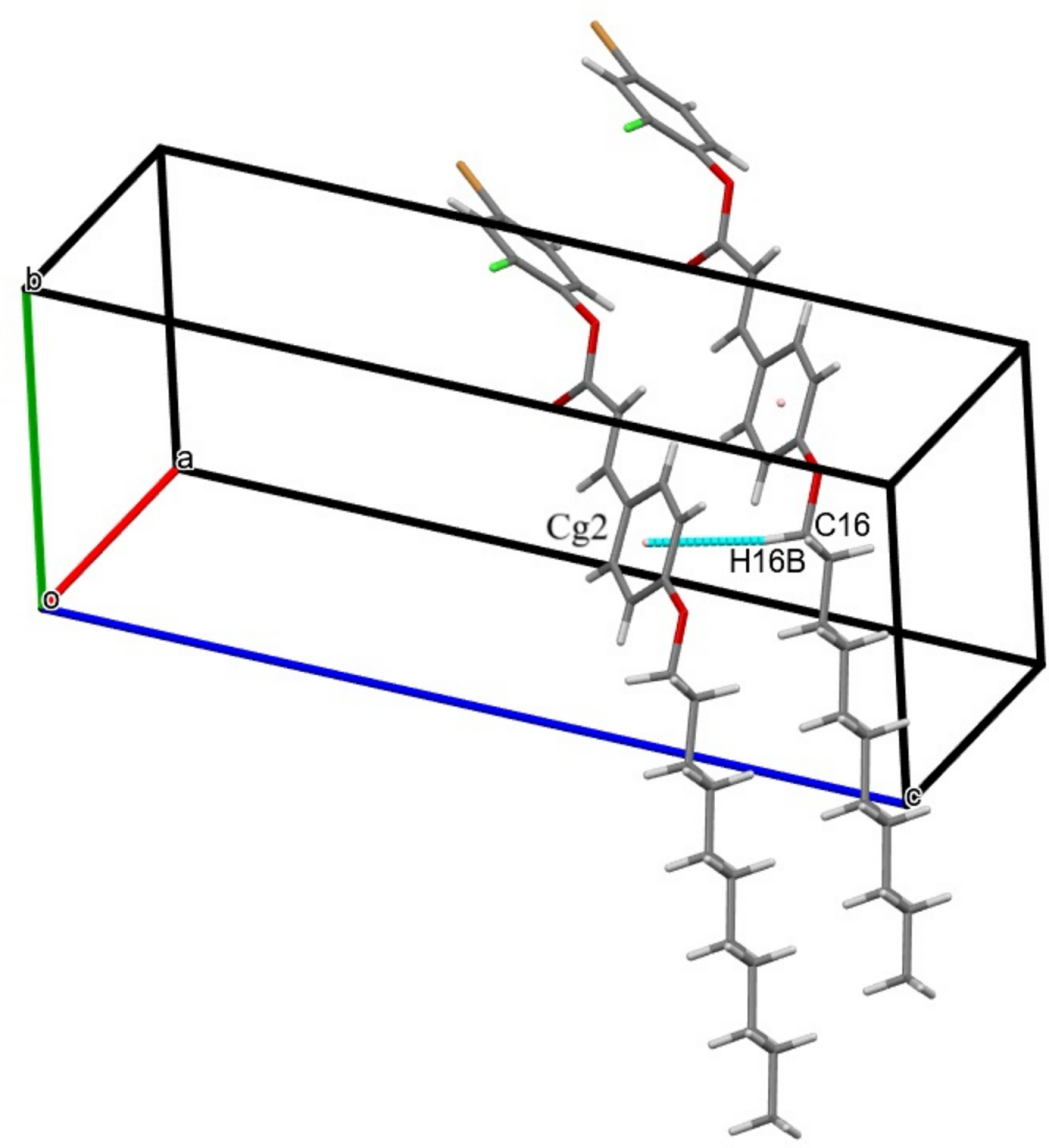
The crystal packing with the C—H⋯π inter­actions depicted by dashed pale-green lines. *Cg*2 is the centroid of the (C10–C15)(*x* + 1, *y*, *z*) ring.

**Figure 4:**
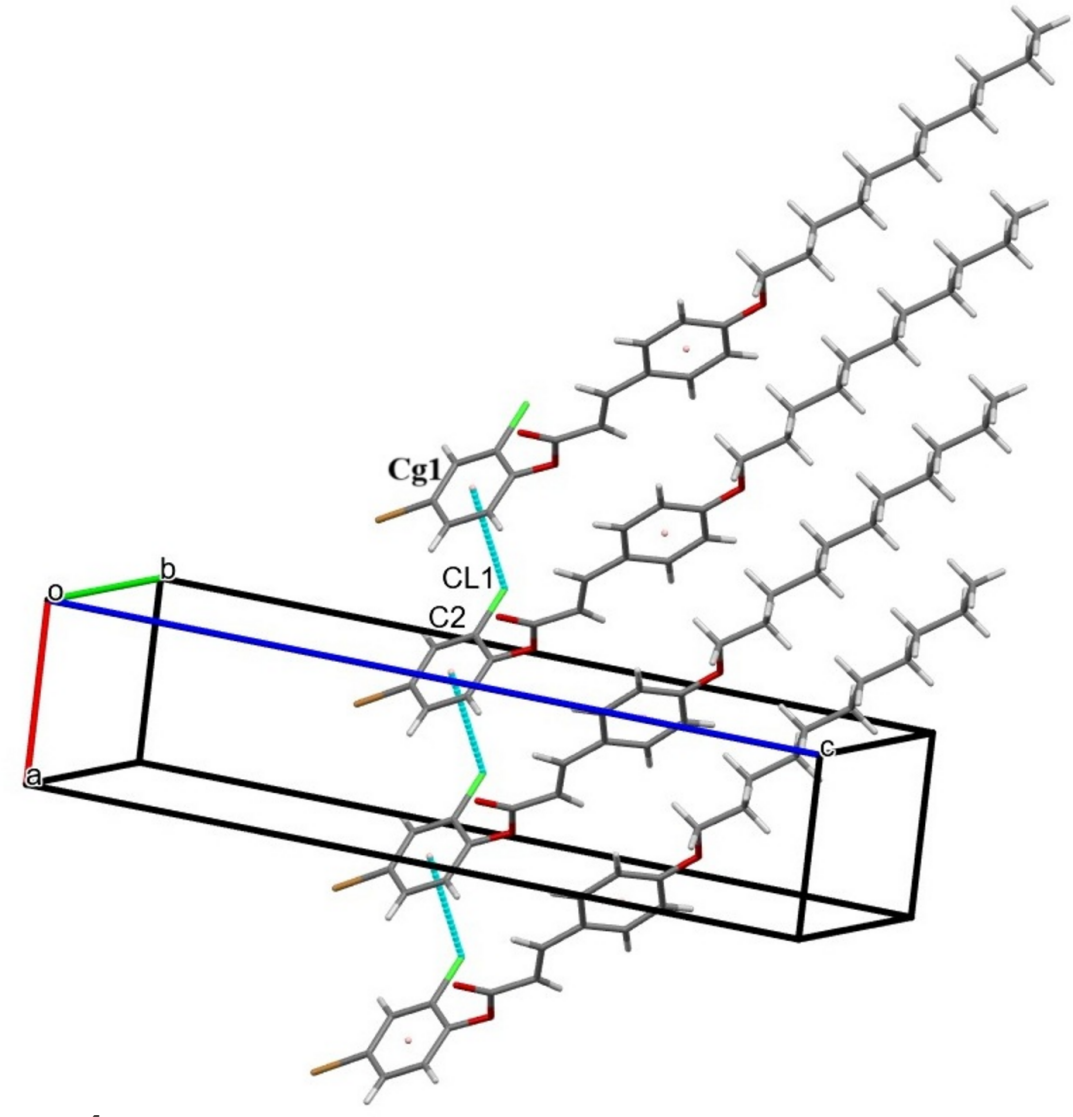
The crystal packing with weak C—Cl⋯*Cg*1 inter­actions depicted by dashed pale-green lines. *Cg*1 is the centroid of the C1–C6 ring.

In the crystal, C—H⋯O hydrogen bonding links the mol­ecules into cyclic hydrogen-bonded inversion dimers with 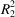
(10) motifs (Table 1 ▸, Fig. 2 ▸). The crystal packing is further consolidated by weak C—H⋯π inter­actions (Table 1 ▸, Fig. 3 ▸). In addition, a weak non-covalent C—Cl⋯π halogen⋯π inter­action arising from the electrostatic attraction between the electron-deficient chlorine atom and the electron-rich π-system connects the mol­ecules along the *a*-axis direction [Cl⋯*Cg*1 distance = 3.6415 (15) Å; *Cg*1 is the centroid for the 4-bromo-2-chloro­phenyl ring (C1–C6); Fig. 4 ▸].

## 4. Hirshfeld surface analysis

**Figure 5:**
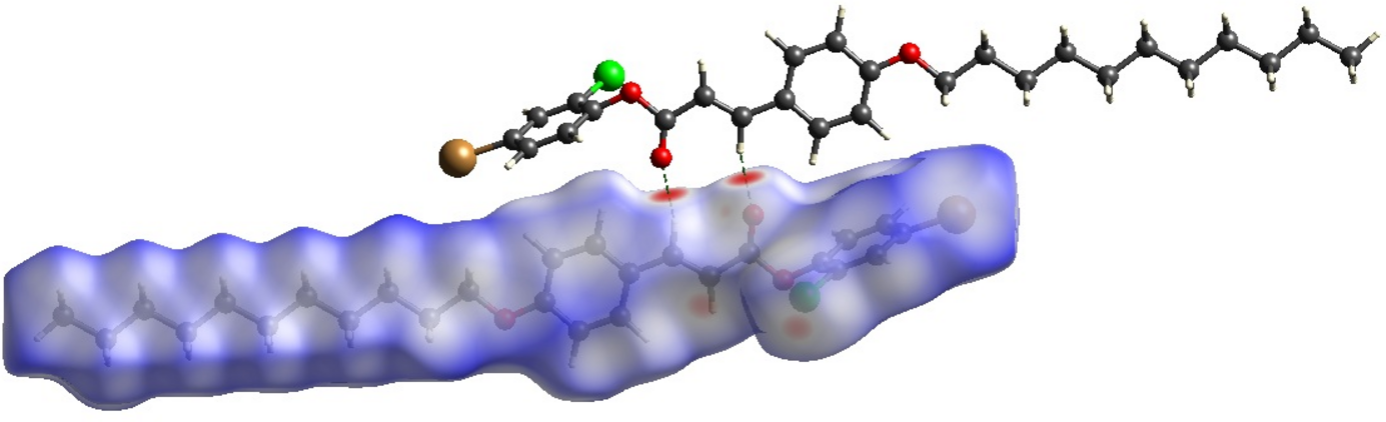
The Hirshfeld surface of the title compound plotted over *d*_norm_ with dashed lines indicating hydrogen bonds.

**Figure 6:**
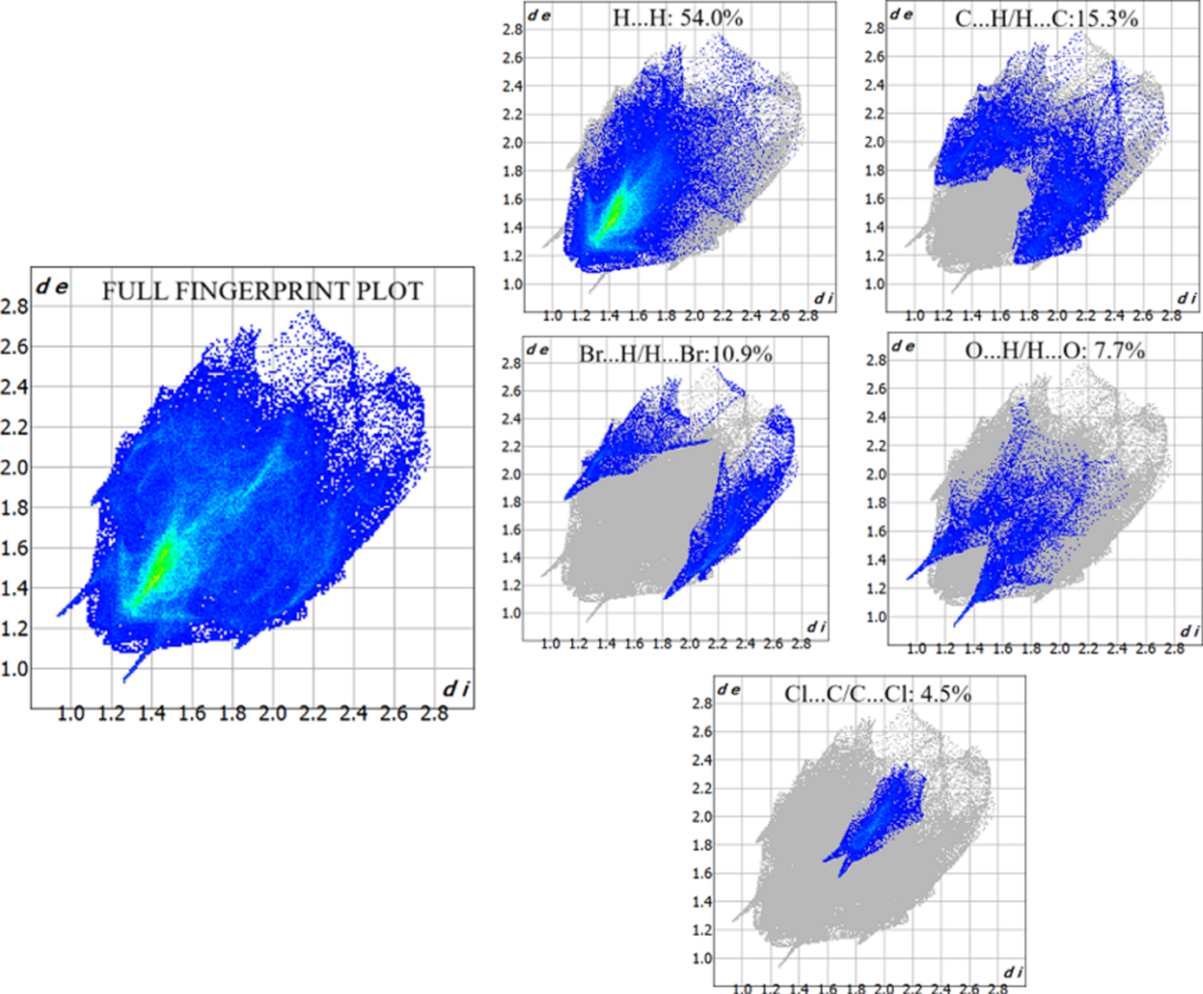
The two-dimensional fingerprint plots for the title compound, showing all inter­actions, and delineated into H⋯H, C⋯H/H⋯C, Br⋯H/H⋯Br, O⋯H/H⋯O, Cl⋯C/C⋯Cl, Cl⋯H/H⋯Cl, Cl⋯O/O⋯Cl, O⋯C/C⋯O, Cl⋯Cl, C⋯C, O⋯O and Br⋯C/C⋯Br inter­actions.

The Hirshfeld surface (Spackman & Jayatilaka, 2009 ▸) and two-dimensional fingerprint plots (McKinnon *et al.*, 2007 ▸) were generated using *CrystalExplorer17* (Spackman *et al.*, 2021 ▸) to investigate the inter­molecular inter­actions. The Hirshfeld surface mapped over *d*_norm_ is shown in Fig. 5 ▸. The prominent red spots on the iso-surface indicate the presence of significant inter­molecular hydrogen-bond inter­actions, specifically C9—H9⋯O2, which play a stabilizing role in the crystal packing. The corresponding 2D fingerprint plots are shown in Fig. 6 ▸, qu­anti­fying the contribution of various inter­molecular inter­actions to the overall crystal packing. The most significant contributions arise from H⋯H (54.0%), followed by C⋯H/H⋯C (15.3%), Br⋯H/H⋯Br (10.9%), O⋯H/H⋯O (7.7%) and Cl⋯H/H⋯Cl (4.5%) contacts. The sharp spikes in the 2D fingerprint plots correspond to the C9—H9⋯O2 hydrogen bond (O⋯H/H⋯O).

## 5. Database Survey

A search of the Cambridge Structural Database (CSD version 2.0.4, December 2024; Groom *et al.*, 2016 ▸) for mol­ecules containing the 4-bromo-2-chloro­phenyl moiety resulted in 15 matches. Among these, the five compounds with CSD codes EBEPUZ (Lehmler *et al.*, 2013 ▸), ISOJUX (Koti Reddy *et al.*, 2016 ▸), FANFOS (Sangeeta *et al.*, 2017 ▸), VIDQUX (Mohan *et al.*, 2018 ▸) and EJULUT (Dumitrescu *et al.*, 2020 ▸), are found to be substituted with fragments containing alk­yloxy chains or substituted aromatic or heterocyclic rings that are in same plane. The dihedral angles between these planes and the 4-bromo-2-chloro­phenyl moiety are 59.0, 75.3, 88.78, 37.47, and 2.99°. In the title compound, the dihedral angle between the 4-bromo-2-chloro­phenyl ring and the planar (undec­yloxy)phen­yl)acrylate fragment is found to be 74.23 (3)°. The torsion angle between the *ortho*-substituted chlorine atom and the first atom of the planar side chain in the above compounds is between 1 to 4° whereas in the title compound it is 5.30 (4)°.

## 6. Synthesis and crystallization

A mixture of 4-bromo-2-chloro­phenol (0.208 g, 0.001 mol) and (*E*)-3-[4-(undec­yloxy)phen­yl]acrylic acid (0.319 g, 0.001 mol) was suspended in anhydrous chloro­form (10 ml). To this, *N*,*N*-di­cyclo­hexyl­carbodi­imide (0.206 g, 0.001 mol) and 4-*N*,*N*-di­methyl­amino pyridine (5 mg) was added and the mixture stirred overnight at room temperature.
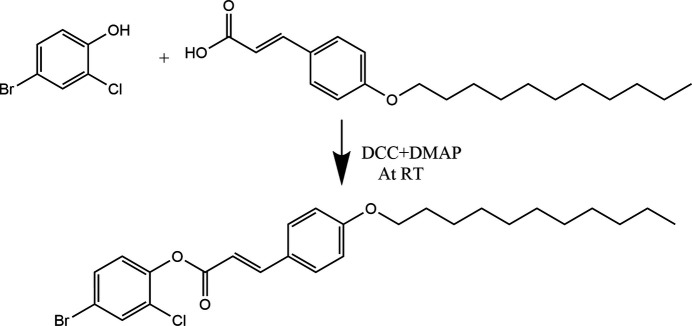


The *N*,*N*-di­cyclo­hexyl urea formed was filtered off and the filtrate diluted with chloro­form (25 ml). This solution was washed successively with 5% aqueous acetic acid solution (2 × 25 ml) and water (2 × 25 ml) and dried on sodium sulfate. The residue obtained on removal of solvent was chromatographed on silica gel using chloro­form as eluent. Removal of solvent from the eluate afforded a white material, which was crystallized from a chloro­form–petroleum ether mixture. Yield (0.385 g, 73%), m.p. 338–340 K. Elemental analysis, calculated: C, 61.49; H, 6.35; Br, 15.73; Cl, 6.98; O, 9.45%, found: C, 61.52; H, 6.38; Br, 15.76; Cl, 6.95%.

## 7. Refinement details

Crystal data, data collection and structure refinement details are summarized in Table 2 ▸. H atoms were positioned geometrically (C—H = 0.93–0.97 Å) and refined using a riding model with *U*_iso_(H) = k*U_eq_(C), where *k*= 1.5 for methyl hydrogen atoms and 1.2 for all others.

## Supplementary Material

Crystal structure: contains datablock(s) I. DOI: 10.1107/S2056989025007078/ee2016sup1.cif

Supporting information file. DOI: 10.1107/S2056989025007078/ee2016Isup3.cml

Structure factors: contains datablock(s) I. DOI: 10.1107/S2056989025007078/ee2016Isup3.hkl

CCDC reference: 2478544

Additional supporting information:  crystallographic information; 3D view; checkCIF report

## Figures and Tables

**Table 1:** Hydrogen-bond geometry (Å, °) *Cg*2 is the centroid of the C10–C15 ring.

*D*—H⋯*A*	*D*—H	H⋯*A*	*D*⋯*A*	*D*—H⋯*A*
C9—H9⋯O2^i^	0.93	2.35	3.248 (3)	164
C16—H16*A*⋯*Cg*2^ii^	0.97	2.95	3.824 (3)	150

Symmetry codes: (i) 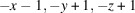; (ii) 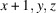.

**Table 2:** Experimental details

Crystal data	
Chemical formula	C_26_H_32_BrClO_3_
*M*_r_	507.87
Crystal system, space group	Triclinic, *P* 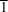
Temperature (K)	296
*a*, *b*, *c* (Å)	5.5111 (8), 9.5856 (15), 23.532 (4)
α, β, γ (°)	81.251 (4), 86.986 (4), 88.012 (4)
*V* (Å^3^)	1226.5 (3)
*Z*	2
Radiation type	Mo *K*α
μ (mm^−1^)	1.81
Crystal size (mm)	0.27 × 0.24 × 0.20

Data collection	
Diffractometer	Bruker SMART APEXII CCD
Absorption correction	Multi-scan (*SADABS*; Krause *et al.*, 2015 ▸)
*T*_min_, *T*_max_	0.6, 0.7
No. of measured, independent and observed [*I* > 2σ(*I*)] reflections	17380, 6085, 5080
*R*_int_	0.051
(sin θ/λ)_max_ (Å^−1^)	0.669

Refinement	
*R*[*F*^2^ > 2σ(*F*^2^)], *wR*(*F*^2^), *S*	0.048, 0.114, 1.09
No. of reflections	6085
No. of parameters	281
H-atom treatment	H-atom parameters constrained
Δρ_max_, Δρ_min_ (e Å^−3^)	0.90, −0.96

Computer programs: *APEX2* and *SAINT* (Bruker, 2014 ▸), *SHELXT* (Sheldrick, 2015*a* ▸), *SHELXL2019/2* (Sheldrick, 2015*b* ▸), *Mercury* (Macrae *et al.*, 2020 ▸) and *publCIF* (Westrip,2010 ▸).

## supplementary crystallographic information

### 4-Bromo-2-chlorophenyl (E)-3-[4-(undecyloxy)phenyl]prop-2-enoate Crystal data

**Table:**

C_26_H_32_BrClO_3_	*Z* = 2
*M**_r_* = 507.87	*F*(000) = 528
Triclinic, *P*1	*D*_x_ = 1.375 Mg m^−^^3^
*a* = 5.5111 (8) Å	Mo *K*α radiation, λ = 0.71073 Å
*b* = 9.5856 (15) Å	Cell parameters from 5080 reflections
*c* = 23.532 (4) Å	θ = 2–28.4°
α = 81.251 (4)°	µ = 1.81 mm^−^^1^
β = 86.986 (4)°	*T* = 296 K
γ = 88.012 (4)°	Prism, colourless
*V* = 1226.5 (3) Å^3^	0.27 × 0.24 × 0.20 mm

### 4-Bromo-2-chlorophenyl (E)-3-[4-(undecyloxy)phenyl]prop-2-enoate Data collection

**Table:**

Bruker SMART APEXII CCD diffractometer	6085 independent reflections
Radiation source: fine-focus sealed tube	5080 reflections with *I* > 2σ(*I*)
Graphite monochromator	*R*_int_ = 0.051
Detector resolution: 1.02 pixels mm^-1^	θ_max_ = 28.4°, θ_min_ = 2.6°
φ and Ω scans	*h* = −7→7
Absorption correction: multi-scan (SADABS; Krause *et al.*, 2015)	*k* = −12→12
*T*_min_ = 0.6, *T*_max_ = 0.7	*l* = −31→31
17380 measured reflections	

### 4-Bromo-2-chlorophenyl (E)-3-[4-(undecyloxy)phenyl]prop-2-enoate Refinement

**Table:**

Refinement on *F*^2^	Primary atom site location: structure-invariant direct methods
Least-squares matrix: full	Secondary atom site location: difference Fourier map
*R*[*F*^2^ > 2σ(*F*^2^)] = 0.048	Hydrogen site location: inferred from neighbouring sites
*wR*(*F*^2^) = 0.114	H-atom parameters constrained
*S* = 1.09	*w* = 1/[σ^2^(*F*_o_^2^) + (0.0321*P*)^2^ + 1.7821*P*] where *P* = (*F*_o_^2^ + 2*F*_c_^2^)/3
6085 reflections	(Δ/σ)_max_ = 0.001
281 parameters	Δρ_max_ = 0.90 e Å^−^^3^
0 restraints	Δρ_min_ = −0.96 e Å^−^^3^
0 constraints	

### 4-Bromo-2-chlorophenyl (E)-3-[4-(undecyloxy)phenyl]prop-2-enoate Special details

**Table:**

Geometry. All esds (except the esd in the dihedral angle between two l.s. planes) are estimated using the full covariance matrix. The cell esds are taken into account individually in the estimation of esds in distances, angles and torsion angles; correlations between esds in cell parameters are only used when they are defined by crystal symmetry. An approximate (isotropic) treatment of cell esds is used for estimating esds involving l.s. planes.

### 4-Bromo-2-chlorophenyl (E)-3-[4-(undecyloxy)phenyl]prop-2-enoate Fractional atomic coordinates and isotropic or equivalent isotropic displacement parameters (Å^2^)

**Table:**

	*x*	*y*	*z*	*U*_iso_*/*U*_eq_	
Br1	−1.44231 (6)	0.07449 (4)	0.72273 (2)	0.03026 (10)	
Cl1	−0.69598 (12)	−0.02663 (8)	0.56674 (3)	0.02653 (16)	
O2	−0.7521 (3)	0.3939 (2)	0.51095 (8)	0.0204 (4)	
O3	0.2021 (3)	0.4438 (2)	0.21602 (8)	0.0203 (4)	
O1	−0.9273 (4)	0.2016 (2)	0.48915 (8)	0.0224 (4)	
C4	−1.2718 (5)	0.1195 (3)	0.65019 (11)	0.0181 (5)	
C10	−0.2950 (4)	0.4111 (3)	0.35655 (10)	0.0146 (5)	
C12	0.0467 (5)	0.5320 (3)	0.30290 (11)	0.0175 (5)	
H12	0.158485	0.603779	0.298670	0.021*	
C13	0.0446 (4)	0.4401 (3)	0.26272 (10)	0.0157 (5)	
C7	−0.7748 (4)	0.3133 (3)	0.47730 (11)	0.0148 (5)	
C3	−1.0695 (5)	0.0384 (3)	0.63777 (11)	0.0196 (5)	
H3	−1.013572	−0.035248	0.664720	0.024*	
C5	−1.3567 (5)	0.2311 (3)	0.61188 (11)	0.0186 (5)	
H5	−1.490918	0.285355	0.621850	0.022*	
C1	−1.0382 (5)	0.1795 (3)	0.54418 (11)	0.0169 (5)	
C8	−0.6485 (4)	0.3140 (3)	0.42096 (11)	0.0159 (5)	
H8	−0.697304	0.256542	0.395425	0.019*	
C11	−0.1211 (4)	0.5154 (3)	0.34965 (11)	0.0160 (5)	
H11	−0.117004	0.575513	0.377070	0.019*	
C2	−0.9519 (5)	0.0700 (3)	0.58382 (11)	0.0177 (5)	
C17	0.5323 (5)	0.5294 (3)	0.15377 (11)	0.0180 (5)	
H17A	0.429058	0.523176	0.122165	0.022*	
H17B	0.620095	0.439804	0.162346	0.022*	
C6	−1.2378 (5)	0.2615 (3)	0.55788 (11)	0.0189 (5)	
H6	−1.292326	0.336328	0.531272	0.023*	
C15	−0.2948 (5)	0.3201 (3)	0.31520 (11)	0.0174 (5)	
H15	−0.409570	0.250043	0.318702	0.021*	
C16	0.3751 (5)	0.5544 (3)	0.20594 (11)	0.0184 (5)	
H16A	0.473556	0.551762	0.239077	0.022*	
H16B	0.292172	0.645838	0.198952	0.022*	
C18	0.7136 (5)	0.6456 (3)	0.13518 (11)	0.0186 (5)	
H18A	0.813274	0.654416	0.167141	0.022*	
H18B	0.626185	0.734666	0.124933	0.022*	
C9	−0.4608 (4)	0.3998 (3)	0.40721 (10)	0.0153 (5)	
H9	−0.432562	0.460077	0.433465	0.018*	
C19	0.8772 (5)	0.6149 (3)	0.08386 (11)	0.0182 (5)	
H19A	0.971302	0.528723	0.095216	0.022*	
H19B	0.775702	0.598798	0.053172	0.022*	
C23	1.5469 (5)	0.7803 (3)	−0.06600 (11)	0.0187 (5)	
H23A	1.640127	0.694139	−0.054020	0.022*	
H23B	1.443821	0.762871	−0.096192	0.022*	
C20	1.0514 (5)	0.7326 (3)	0.06060 (11)	0.0185 (5)	
H20A	1.154647	0.748415	0.091027	0.022*	
H20B	0.958141	0.819163	0.049260	0.022*	
C14	−0.1262 (5)	0.3332 (3)	0.26937 (11)	0.0183 (5)	
H14	−0.125992	0.270754	0.242746	0.022*	
C21	1.2113 (5)	0.6993 (3)	0.00905 (11)	0.0184 (5)	
H21A	1.304137	0.612633	0.020503	0.022*	
H21B	1.107613	0.683149	−0.021230	0.022*	
C25	1.8825 (5)	0.8593 (3)	−0.14085 (12)	0.0217 (6)	
H25A	1.780445	0.842081	−0.171282	0.026*	
H25B	1.974251	0.772814	−0.128449	0.026*	
C24	1.7217 (5)	0.8967 (3)	−0.09043 (11)	0.0189 (5)	
H24A	1.628795	0.982639	−0.102987	0.023*	
H24B	1.823950	0.915000	−0.060221	0.023*	
C22	1.3861 (5)	0.8153 (3)	−0.01485 (11)	0.0189 (5)	
H22A	1.488999	0.832089	0.015483	0.023*	
H22B	1.293413	0.901732	−0.026726	0.023*	
C26	2.0588 (6)	0.9755 (3)	−0.16466 (13)	0.0295 (7)	
H26A	2.157249	0.946694	−0.195930	0.044*	
H26B	2.161360	0.992456	−0.134811	0.044*	
H26C	1.968879	1.060577	−0.178209	0.044*	

### 4-Bromo-2-chlorophenyl (E)-3-[4-(undecyloxy)phenyl]prop-2-enoate Atomic displacement parameters (Å^2^)

**Table:**

	*U*^11^	*U*^22^	*U*^33^	*U*^12^	*U*^13^	*U*^23^
Br1	0.03308 (17)	0.03720 (19)	0.01798 (14)	−0.00773 (13)	0.01127 (11)	0.00119 (11)
Cl1	0.0175 (3)	0.0268 (4)	0.0378 (4)	0.0014 (3)	0.0032 (3)	−0.0148 (3)
O2	0.0206 (9)	0.0218 (10)	0.0197 (9)	−0.0075 (8)	0.0081 (7)	−0.0078 (8)
O3	0.0223 (9)	0.0216 (10)	0.0170 (9)	−0.0089 (8)	0.0112 (7)	−0.0049 (7)
O1	0.0266 (10)	0.0254 (10)	0.0166 (9)	−0.0153 (8)	0.0099 (8)	−0.0076 (8)
C4	0.0214 (12)	0.0197 (13)	0.0129 (11)	−0.0079 (10)	0.0054 (9)	−0.0020 (9)
C10	0.0142 (11)	0.0148 (12)	0.0133 (11)	−0.0002 (9)	0.0035 (9)	0.0004 (9)
C12	0.0159 (11)	0.0174 (12)	0.0194 (12)	−0.0053 (10)	0.0035 (10)	−0.0034 (10)
C13	0.0156 (11)	0.0175 (12)	0.0131 (11)	−0.0010 (10)	0.0042 (9)	−0.0008 (9)
C7	0.0107 (10)	0.0150 (12)	0.0183 (12)	−0.0017 (9)	0.0005 (9)	−0.0014 (9)
C3	0.0218 (12)	0.0174 (13)	0.0187 (12)	−0.0033 (10)	−0.0010 (10)	0.0011 (10)
C5	0.0163 (11)	0.0194 (13)	0.0199 (12)	−0.0019 (10)	0.0038 (10)	−0.0031 (10)
C1	0.0172 (11)	0.0185 (12)	0.0155 (12)	−0.0089 (10)	0.0054 (9)	−0.0043 (10)
C8	0.0157 (11)	0.0181 (12)	0.0140 (11)	−0.0012 (10)	0.0015 (9)	−0.0033 (9)
C11	0.0156 (11)	0.0163 (12)	0.0163 (11)	−0.0001 (10)	0.0023 (9)	−0.0042 (9)
C2	0.0146 (11)	0.0162 (12)	0.0232 (13)	−0.0027 (10)	0.0024 (10)	−0.0065 (10)
C17	0.0177 (11)	0.0213 (13)	0.0146 (11)	−0.0040 (10)	0.0050 (9)	−0.0028 (10)
C6	0.0204 (12)	0.0171 (12)	0.0176 (12)	−0.0038 (10)	0.0012 (10)	0.0029 (10)
C15	0.0201 (12)	0.0158 (12)	0.0162 (12)	−0.0058 (10)	0.0034 (10)	−0.0017 (9)
C16	0.0193 (12)	0.0185 (13)	0.0173 (12)	−0.0060 (10)	0.0054 (10)	−0.0031 (10)
C18	0.0167 (11)	0.0202 (13)	0.0185 (12)	−0.0050 (10)	0.0067 (10)	−0.0030 (10)
C9	0.0150 (11)	0.0157 (12)	0.0143 (11)	−0.0001 (9)	0.0021 (9)	−0.0007 (9)
C19	0.0176 (11)	0.0201 (13)	0.0167 (12)	−0.0038 (10)	0.0064 (10)	−0.0036 (10)
C23	0.0184 (12)	0.0169 (12)	0.0203 (13)	−0.0034 (10)	0.0068 (10)	−0.0032 (10)
C20	0.0148 (11)	0.0191 (13)	0.0213 (13)	−0.0045 (10)	0.0059 (10)	−0.0038 (10)
C14	0.0224 (12)	0.0167 (12)	0.0161 (12)	−0.0056 (10)	0.0055 (10)	−0.0047 (10)
C21	0.0173 (11)	0.0181 (13)	0.0194 (12)	−0.0046 (10)	0.0071 (10)	−0.0037 (10)
C25	0.0227 (13)	0.0212 (13)	0.0208 (13)	−0.0072 (11)	0.0102 (10)	−0.0039 (10)
C24	0.0183 (12)	0.0188 (13)	0.0197 (12)	−0.0054 (10)	0.0058 (10)	−0.0041 (10)
C22	0.0182 (12)	0.0194 (13)	0.0189 (12)	−0.0050 (10)	0.0064 (10)	−0.0040 (10)
C26	0.0307 (15)	0.0310 (16)	0.0262 (14)	−0.0122 (13)	0.0154 (12)	−0.0054 (12)

### 4-Bromo-2-chlorophenyl (E)-3-[4-(undecyloxy)phenyl]prop-2-enoate Geometric parameters (Å, º)

**Table:**

Br1—C4	1.902 (2)	C15—H15	0.9300
Cl1—C2	1.724 (3)	C16—H16A	0.9700
O2—C7	1.201 (3)	C16—H16B	0.9700
O3—C13	1.361 (3)	C18—C19	1.527 (3)
O3—C16	1.435 (3)	C18—H18A	0.9700
O1—C7	1.370 (3)	C18—H18B	0.9700
O1—C1	1.392 (3)	C9—H9	0.9300
C4—C5	1.376 (4)	C19—C20	1.525 (3)
C4—C3	1.381 (4)	C19—H19A	0.9700
C10—C11	1.395 (3)	C19—H19B	0.9700
C10—C15	1.402 (3)	C23—C24	1.524 (3)
C10—C9	1.456 (3)	C23—C22	1.529 (3)
C12—C13	1.388 (4)	C23—H23A	0.9700
C12—C11	1.393 (3)	C23—H23B	0.9700
C12—H12	0.9300	C20—C21	1.529 (3)
C13—C14	1.401 (3)	C20—H20A	0.9700
C7—C8	1.464 (3)	C20—H20B	0.9700
C3—C2	1.391 (4)	C14—H14	0.9300
C3—H3	0.9300	C21—C22	1.519 (3)
C5—C6	1.393 (3)	C21—H21A	0.9700
C5—H5	0.9300	C21—H21B	0.9700
C1—C6	1.383 (4)	C25—C24	1.523 (3)
C1—C2	1.383 (4)	C25—C26	1.526 (4)
C8—C9	1.339 (3)	C25—H25A	0.9700
C8—H8	0.9300	C25—H25B	0.9700
C11—H11	0.9300	C24—H24A	0.9700
C17—C16	1.510 (3)	C24—H24B	0.9700
C17—C18	1.521 (3)	C22—H22A	0.9700
C17—H17A	0.9700	C22—H22B	0.9700
C17—H17B	0.9700	C26—H26A	0.9600
C6—H6	0.9300	C26—H26B	0.9600
C15—C14	1.380 (3)	C26—H26C	0.9600
			
C13—O3—C16	117.9 (2)	C19—C18—H18B	109.3
C7—O1—C1	116.30 (19)	H18A—C18—H18B	107.9
C5—C4—C3	122.6 (2)	C8—C9—C10	128.5 (2)
C5—C4—Br1	118.51 (19)	C8—C9—H9	115.7
C3—C4—Br1	118.9 (2)	C10—C9—H9	115.7
C11—C10—C15	118.0 (2)	C20—C19—C18	114.1 (2)
C11—C10—C9	118.2 (2)	C20—C19—H19A	108.7
C15—C10—C9	123.8 (2)	C18—C19—H19A	108.7
C13—C12—C11	119.1 (2)	C20—C19—H19B	108.7
C13—C12—H12	120.5	C18—C19—H19B	108.7
C11—C12—H12	120.5	H19A—C19—H19B	107.6
O3—C13—C12	124.5 (2)	C24—C23—C22	113.6 (2)
O3—C13—C14	115.6 (2)	C24—C23—H23A	108.8
C12—C13—C14	119.8 (2)	C22—C23—H23A	108.8
O2—C7—O1	121.8 (2)	C24—C23—H23B	108.8
O2—C7—C8	127.6 (2)	C22—C23—H23B	108.8
O1—C7—C8	110.5 (2)	H23A—C23—H23B	107.7
C4—C3—C2	118.1 (2)	C19—C20—C21	112.8 (2)
C4—C3—H3	120.9	C19—C20—H20A	109.0
C2—C3—H3	120.9	C21—C20—H20A	109.0
C4—C5—C6	118.8 (2)	C19—C20—H20B	109.0
C4—C5—H5	120.6	C21—C20—H20B	109.0
C6—C5—H5	120.6	H20A—C20—H20B	107.8
C6—C1—C2	120.9 (2)	C15—C14—C13	120.4 (2)
C6—C1—O1	120.4 (2)	C15—C14—H14	119.8
C2—C1—O1	118.7 (2)	C13—C14—H14	119.8
C9—C8—C7	118.1 (2)	C22—C21—C20	113.7 (2)
C9—C8—H8	120.9	C22—C21—H21A	108.8
C7—C8—H8	120.9	C20—C21—H21A	108.8
C12—C11—C10	121.9 (2)	C22—C21—H21B	108.8
C12—C11—H11	119.0	C20—C21—H21B	108.8
C10—C11—H11	119.0	H21A—C21—H21B	107.7
C1—C2—C3	120.2 (2)	C24—C25—C26	112.6 (2)
C1—C2—Cl1	120.1 (2)	C24—C25—H25A	109.1
C3—C2—Cl1	119.8 (2)	C26—C25—H25A	109.1
C16—C17—C18	112.7 (2)	C24—C25—H25B	109.1
C16—C17—H17A	109.1	C26—C25—H25B	109.1
C18—C17—H17A	109.1	H25A—C25—H25B	107.8
C16—C17—H17B	109.1	C25—C24—C23	112.9 (2)
C18—C17—H17B	109.1	C25—C24—H24A	109.0
H17A—C17—H17B	107.8	C23—C24—H24A	109.0
C1—C6—C5	119.5 (2)	C25—C24—H24B	109.0
C1—C6—H6	120.3	C23—C24—H24B	109.0
C5—C6—H6	120.3	H24A—C24—H24B	107.8
C14—C15—C10	120.8 (2)	C21—C22—C23	113.3 (2)
C14—C15—H15	119.6	C21—C22—H22A	108.9
C10—C15—H15	119.6	C23—C22—H22A	108.9
O3—C16—C17	106.8 (2)	C21—C22—H22B	108.9
O3—C16—H16A	110.4	C23—C22—H22B	108.9
C17—C16—H16A	110.4	H22A—C22—H22B	107.7
O3—C16—H16B	110.4	C25—C26—H26A	109.5
C17—C16—H16B	110.4	C25—C26—H26B	109.5
H16A—C16—H16B	108.6	H26A—C26—H26B	109.5
C17—C18—C19	111.7 (2)	C25—C26—H26C	109.5
C17—C18—H18A	109.3	H26A—C26—H26C	109.5
C19—C18—H18A	109.3	H26B—C26—H26C	109.5
C17—C18—H18B	109.3		
			
C16—O3—C13—C12	−3.1 (4)	C4—C3—C2—Cl1	179.0 (2)
C16—O3—C13—C14	177.7 (2)	C2—C1—C6—C5	−1.5 (4)
C11—C12—C13—O3	−178.6 (2)	O1—C1—C6—C5	175.5 (2)
C11—C12—C13—C14	0.5 (4)	C4—C5—C6—C1	−0.2 (4)
C1—O1—C7—O2	−5.4 (4)	C11—C10—C15—C14	0.4 (4)
C1—O1—C7—C8	173.1 (2)	C9—C10—C15—C14	−177.4 (2)
C5—C4—C3—C2	−1.4 (4)	C13—O3—C16—C17	177.4 (2)
Br1—C4—C3—C2	178.0 (2)	C18—C17—C16—O3	175.7 (2)
C3—C4—C5—C6	1.7 (4)	C16—C17—C18—C19	177.7 (2)
Br1—C4—C5—C6	−177.7 (2)	C7—C8—C9—C10	174.4 (2)
C7—O1—C1—C6	78.9 (3)	C11—C10—C9—C8	175.9 (3)
C7—O1—C1—C2	−104.1 (3)	C15—C10—C9—C8	−6.3 (4)
O2—C7—C8—C9	10.5 (4)	C17—C18—C19—C20	176.0 (2)
O1—C7—C8—C9	−167.9 (2)	C18—C19—C20—C21	−179.6 (2)
C13—C12—C11—C10	−1.6 (4)	C10—C15—C14—C13	−1.4 (4)
C15—C10—C11—C12	1.1 (4)	O3—C13—C14—C15	−179.9 (2)
C9—C10—C11—C12	179.0 (2)	C12—C13—C14—C15	0.9 (4)
C6—C1—C2—C3	1.8 (4)	C19—C20—C21—C22	179.9 (2)
O1—C1—C2—C3	−175.3 (2)	C26—C25—C24—C23	−179.4 (2)
C6—C1—C2—Cl1	−177.6 (2)	C22—C23—C24—C25	179.3 (2)
O1—C1—C2—Cl1	5.4 (3)	C20—C21—C22—C23	179.4 (2)
C4—C3—C2—C1	−0.3 (4)	C24—C23—C22—C21	179.6 (2)

### 4-Bromo-2-chlorophenyl (E)-3-[4-(undecyloxy)phenyl]prop-2-enoate Hydrogen-bond geometry (Å, º)

*Cg*2 is the centroid of the C10–C15 ring.

**Table:**

*D*—H···*A*	*D*—H	H···*A*	*D*···*A*	*D*—H···*A*
C9—H9···O2^i^	0.93	2.35	3.248 (3)	164
C16—H16*A*···*Cg*2^ii^	0.97	2.95	3.824 (3)	150

Symmetry codes: (i) −*x*−1, −*y*+1, −*z*+1; (ii) *x*+1, *y*, *z*.
